# Gastrinoma With Relatively Low Gastrin Levels: A Case Report

**DOI:** 10.7759/cureus.41686

**Published:** 2023-07-11

**Authors:** Amelia Winczura, Bob Saggi, David Savage-Lobeck

**Affiliations:** 1 Surgery, Saint James School of Medicine, McAllen, USA; 2 General Surgery, South Texas Health System, McAllen, USA; 3 General Surgery, Saint James School of Medicine, McAllen, USA

**Keywords:** pet scan, octreotide scan, ct scan, proton pump inhibitors, secretin, gastric acid, gastrin levels, neuroendocrine tumors, gastrinoma

## Abstract

Gastrinomas can often be cured with surgical removal if detected early enough. We present a case report where a patient was diagnosed with gastrinoma with relatively low serum gastrin levels after subsequent duodenojejunostomy, gastrojejunostomy, total gastrectomy, and cholecystectomy. With this case report, we hope to promote a higher level of awareness of pancreatic neuroendocrine (NE) tumors and educate clinicians on the full effects of proton pump inhibitor (PPI) use on patient workup, diagnostics, and care.

## Introduction

Gastrinomas are a relatively rare form of neuroendocrine (NE) tumor, with an annual incidence rate of 0.5-2 per million [1-3]. These neoplasms are more common in males compared to females and are typically diagnosed during the second to fifth decade of life [4]. A majority of gastrinomas occur sporadically, and only roughly a quarter of the cases are due to mutation in the multiple endocrine neoplasia type 1 (MEN1) tumor suppressor gene on chromosome 11 [5,6]. This mutation can lead to a collection of NE tumors (NET); pituitary tumors most commonly secrete prolactin, parathyroid tumors secrete parathyroid hormone (PTH) leading to increased ionized calcium levels, and pancreatic tumors commonly secrete gastrin, vasointestinal peptide, insulin, glucagon, or somatostatin [7].

Gastrinomas most commonly occur in the gastrinoma triangle (also known as Passaro’s triangle), formed by the pancreatic head/neck, the confluence of the cystic duct and common hepatic duct, and the second and third parts of the duodenum [8,9]. Some gastrinomas can occur ectopically, outside Passaro’s triangle; the most common location of these neoplasms is the pancreas [8-10]. The differential diagnoses for gastrinomas include retained gastric antrum, gastric outlet obstruction, and antral G cell hyperplasia [10].

Gastric acid is typically secreted by the G cells located within the stomach and pancreas [11]. The excess secretion of gastric acid can cause a multitude of effects and problems in the human body. Increased gastrin levels can lead to an increase in acidity in the stomach and small intestine leading to potential mucosal damage and ulceration, as well as the inability to absorb sodium and water properly [11]. Prolonged undiagnosed ulcers can then lead to perforation, which leads to many other downstream complications such as infection, bleeding, sepsis, and death [11]. The increase in gastric acid levels also causes the inactivation of pancreatic enzymes, which typically help digest fats and proteins, leading to steatorrhea, diarrhea, malnutrition, and weight loss [11].

The best initial test for diagnosing a gastrinoma is an esophagogastroduodenoscopy (EGD), revealing ulcerations and thickened gastric folds, a decrease in gastric pH, and an increase in basal acid output of greater than 15 mEq per hour [2,12,13]. Traditionally, the diagnosis of gastrinoma is confirmed with gastrin levels 10 times the upper limit of normal (typically >1000 pg/mL; normal {N}: 0-100 pg/mL) in the presence of gastric pH of less than 2 [4]. However, it has been noted that many gastrinomas have gastric acid concentrations less than 10 times the upper limit [4]. If gastrin levels are increased, but not more than 1000 pg/mL, it is recommended to follow up with a secretin stimulation test [10]. Normally, secretin inhibits G cells and gastric acid release; however, secretin stimulates the release of gastrin by gastrinoma cells, and the patients will have a dramatic increase in gastrin levels after secretin administration [10]. This test should not be performed in patients who are currently taking proton pump inhibitors (PPIs), as it can affect the results [10]. The secretin stimulation test is achieved by giving the patient 0.4 μg/kg of secretin by rapid infusion intravenously over one minute [10]. Subsequently, serum gastrin levels are measured at two minutes, five minutes, and 10 minutes following the administration of the secretin [10]. An increase in gastrin levels of greater than 120 pg/mL over the fasting basal gastrin levels is indicative of a positive result [10]. This stimulation test should not, however, be done on the patients with severe symptoms of gastrinoma, as it can cause further ulceration and put one at risk for further life-threatening complications [10].

There is a clear need to create more accurate and precise diagnostic methods for the diagnosis of gastrinomas. Immunoassay kits historically vary significantly in their accuracy; a study evaluating 12 different kits showed that 33% of kits returned falsely low results in 20%-80% of the patients [14,15]. Another less commonly used diagnostic test for gastrinoma is the measurement of serum chromogranin A (CgA) levels; however, it is not a specific test for gastrinomas but rather a general marker for NE tumors [10]. The lack of adequacy in diagnostic methods is likely overlooked due to the relative rarity of the diagnosis [10].

Computed tomography (CT) scan or positron emission tomography (PET) scan can also be of use in the diagnostic process. A mass in stereotypical locations for NE neoplasms can be seen, in combination with elevated gastrin levels [10]. Diagnosis can be aided with emerging technologies; endoscopic ultrasound can be used, as well as gallium 68 (^68^GA)-labeled somatostatin radiotracers used with positron emission tomography/computed tomography (^68^Ga-DOTATOC, ^68^Ga-DOTANOC, and ^68^Ga-DOTATATE). Radiolabeled indium-111 octreotide scans can also be done, wherein radiolabeled octreotide is taken up by somatostatin receptor-expressing cells. These receptors are maximally expressed in most well-differentiated NE tumors [10].

Due to the rare number of cases each year, gastrinomas are often underdiagnosed, and those that are caught often progress to the point of distant metastasis (most commonly the liver) [9]. Over 60% of gastrinomas are malignant, with the remaining 40% benign [6]. In one prospective study, liver metastases were found in almost 25% of the patients at the time of diagnoses. The patients with such metastases have only a 30% chance of five-year survival. Those without liver metastases had a 15-year survival rate of 83% [16]. The prognosis of gastrinoma is largely dependent on the tumor’s malignancy status, degree of differentiation, extent of disease involvement, and success of surgical intervention [10,16].

Immunohistochemistry can be done to confirm the diagnosis of NE tumor after the subsequent biopsy or removal of the tumor. Common positive stains for gastrinoma include synaptophysin, chromogranin, gastrin, pancreatic and duodenal homeobox 1 (PDX1), and Insulin-associated protein 1 (INSM1) [17]. Gastrinomas typically stain negative for cytokeratin 7 (CK7), caudal-related homeobox transcription factor 2 (CDX2), B cell lymphoma/leukemia 10 (BCL10), nuclear beta-catenin, carbohydrate antigen 19-9 (CA19-9), carcinoembryonic antigen (CEA), and alpha-fetoprotein (AFP) [17]. Ki67 is an alternative approach for measuring gastrinoma grade; as a marker of cell cycle proliferation, it is commonly used to determine if there are potential distant metastases [9].

## Case presentation

After nine years of multiple ER visits, many surgical consults, gastroenterologist visits, surgical and diagnostic procedures including numerous imaging modalities, and medication administration and changes, a 26-year-old obese Hispanic male was ultimately diagnosed with and received treatment for a gastrinoma.

He originally presented to the ER in 2013 for abdominal pain, nausea, vomiting, and diarrhea. His past medical history at the time of initial symptom onset included diagnoses of type II diabetes mellitus, obesity, hyperlipidemia, and hypertension. He had no significant past surgical history or social history at the time of original symptom onset. His family history was significant for unknown cancer in his mother and brother. This was his first time experiencing these symptoms, and he was subsequently diagnosed with unspecified noninfectious gastroenteritis and discharged home. A timeline of events can be seen in the Appendices.

**Figure 1:**
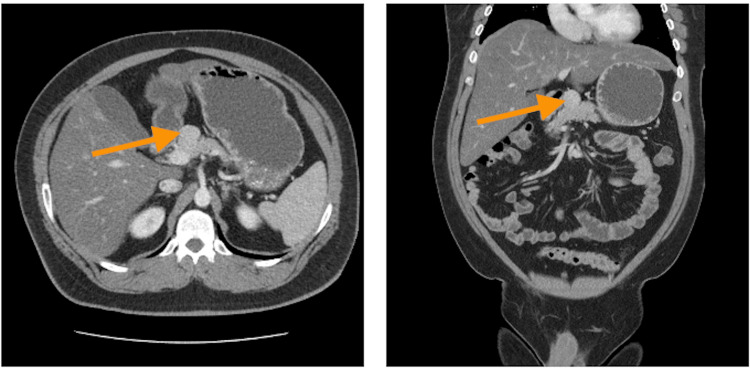
Computed tomography scan of the abdomen with contrast Axial and coronal computed tomography images with contrast are seen above. The orange arrow in each image delineates the tumor location. These images were taken early on in the disease course

Over the course of roughly five years, beginning in 2018, the patient would return to the ED multiple times with similar symptoms, being discharged with a diagnosis of fatty liver disease and gastroenteritis after an abdominopelvic CT series (Figure 1). Later in the year, he returned again with similar symptoms, but this time, his pain was much more severe; the workup was significant for numerous ruptured jejunal ulcers, signs of peritonitis, and severe sepsis. The patient subsequently underwent diagnostic laparoscopy, which was converted to exploratory laparotomy to allow small bowel resection without anastomosis. The patient was left discontinuous, with a laparotomy pad left behind to pack the duodenum; the viability at the duodenal jejunal flexure was questionable. The patient was then taken back to surgery a few days later for the resection of the third and fourth portions of the duodenum due to necrosis, small bowel resection, gastrojejunostomy, pyloroplasty, and the insertion of gastroduodenostomy tube. The pathology reports of specimens showed extensive ischemic and necrotic changes with areas of hemorrhage, findings consistent with necrotizing enteritis with perforation. The details of the case were sent to an outside organization for a second opinion, which diagnosed the patient with perforated necrotizing enteritis.

Later in 2018, the patient returned to surgery for the treatment of severe alkaline reflux gastritis arising from the blind ending duodenum and marginal ulceration with progression to the point of gastric outlet obstruction. Truncal vagotomy with antrectomy was performed, as well as Roux-en-Y gastrojejunostomy, duodenojejunostomy, and cholecystectomy. Slightly over a year after these procedures, in early 2020, the patient began 40 mg of pantoprazole for symptomatic relief. Eight months later, in September, serum gastrin studies were ordered, as symptoms had not yet been abated; the results showed mild hypergastrinemia of 231 pg/mL (N: 0-100 pg/mL). There is documentation that the patient was taking PPIs during this time period. Images taken in November 2020 after these findings showed marginal ulcerations, findings consistent with status post truncal vagotomy and antrectomy. The patient subsequently underwent a near-total gastrectomy for symptomatic relief. The pathology reports of specimens collected during the procedure showed the presence of an ulcer bed with hemorrhage, transmural inflammation, and suggestions of a fissuring ulcer with scattered multinucleated giant cells and fibrous thrombi.

**Figure 2:**
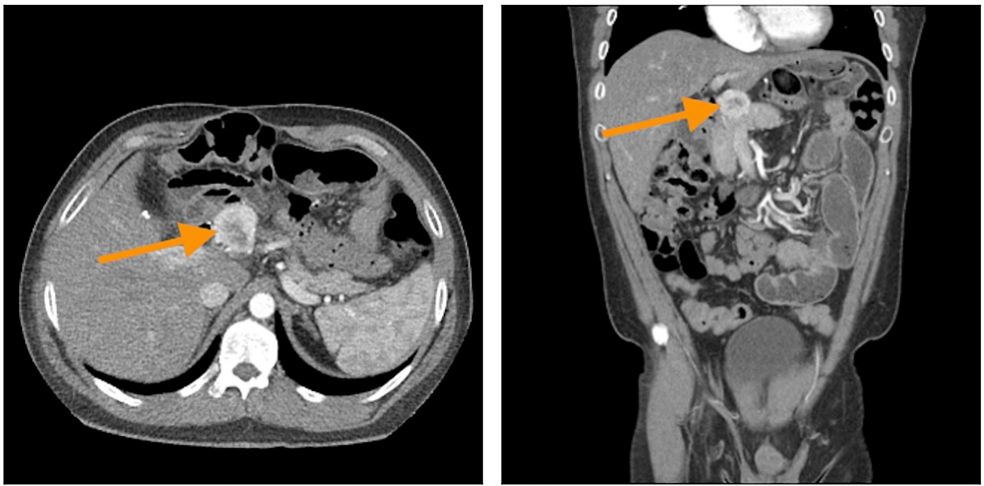
Computed tomography scan of the abdomen with contrast Axial and coronal computed tomography images with contrast are seen above. The orange arrow in each image delineates the tumor location. These images were taken roughly five years after the original symptom onset

The persistence of these symptoms despite intensive medical and surgical interventions raised concern for a gastrinoma, even in the context of only mild hypergastrinemia. In January 2021, the patient’s gastrin levels were measured again, and the results showed a gastrin level of 279 pg/mL. There is documentation that the patient was asked to stop PPI use for two weeks prior to measuring gastrin levels. A CT was performed shortly after gastrin levels were collected, and radiology reported no significant findings. The results of an octreotide scan performed in January 2022 showed attenuation and uptake in the localized area of tumor location without signs of metastasis. In February 2022, another CT was collected at an outside facility, which reported a mass near the caudate lobe of the liver; this can be seen in Figure 2 and was correlated back to the previous scan. Chromogranin A (CgA) levels were also obtained in February 2022, showing levels at 359 ng/mL (N: <95 ng/mL). PET scan in June 2022 showed a well-circumscribed focus of mild hypermetabolism in the periportal region adjacent to surgical sutures, and continuity with the pancreas was indeterminate. No evidence for malignancy was seen.

**Figure 3:**
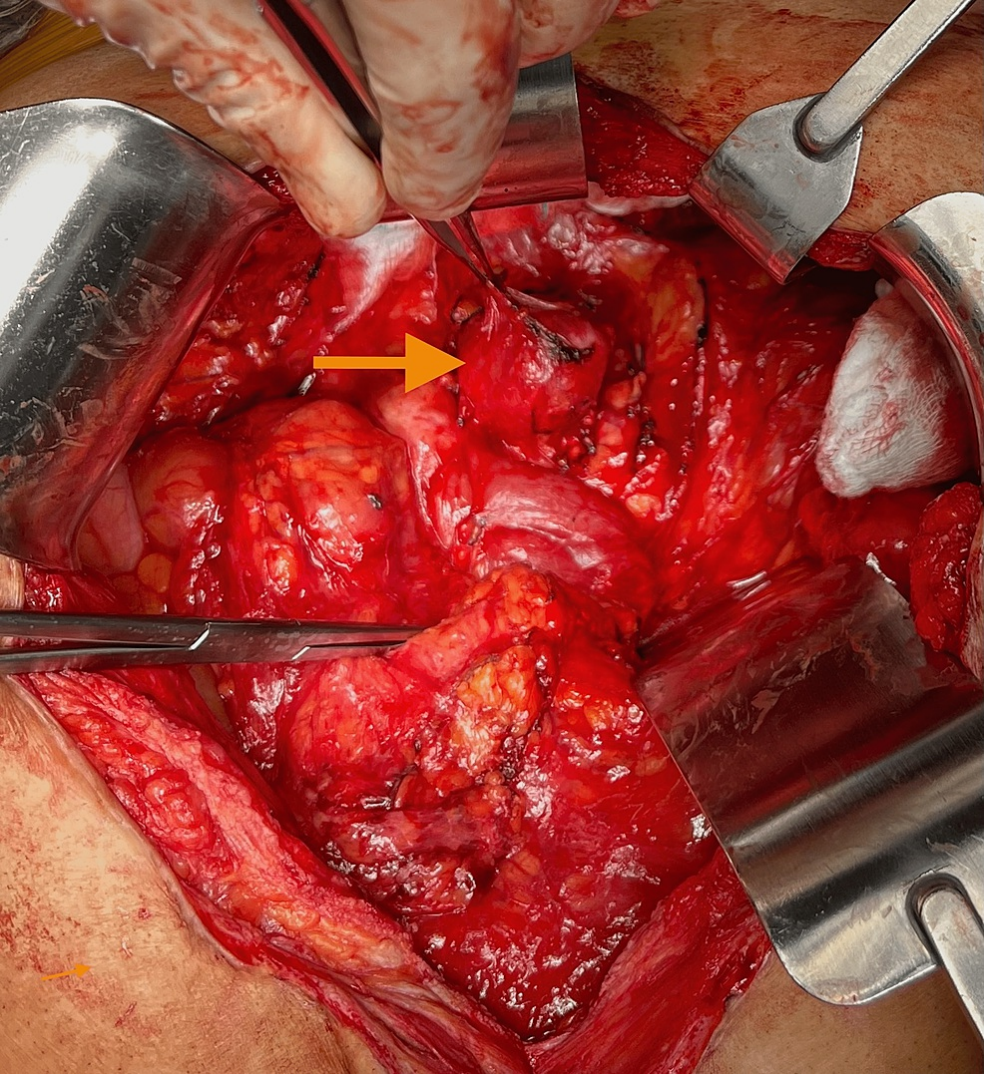
Surgical view of gastrinoma The orange arrow in the image delineates the tumor location

**Figure 4:**
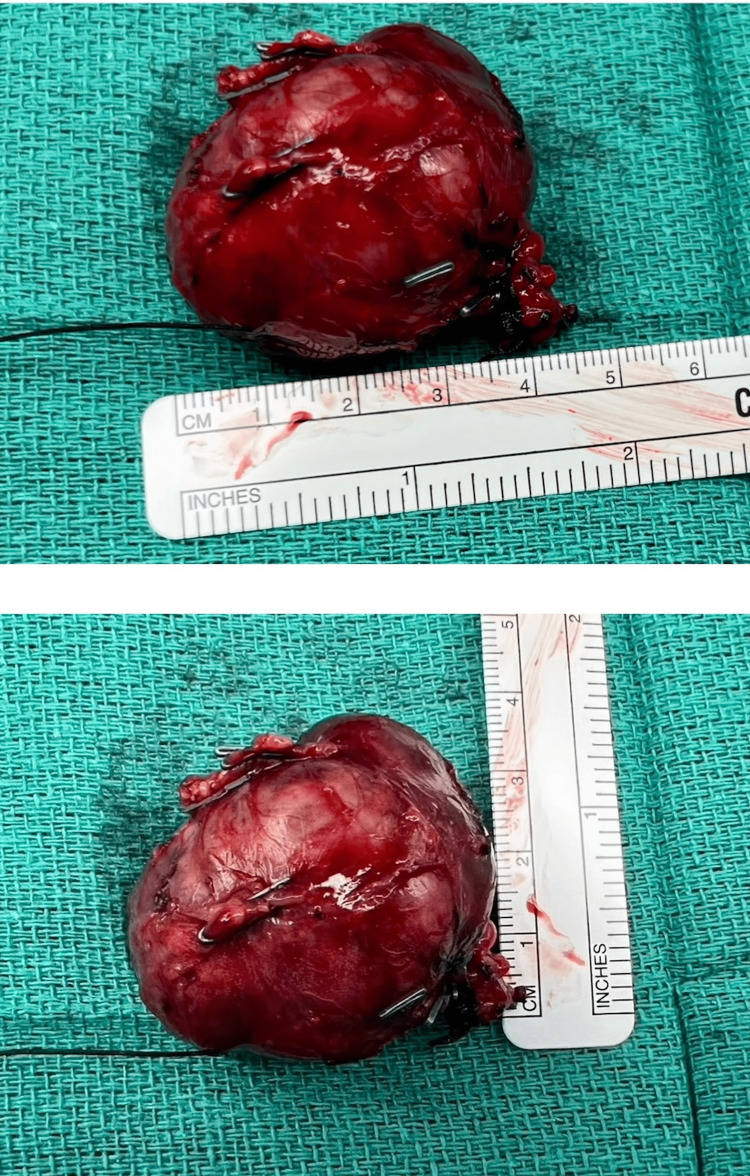
Specimen upon removal from the body cavity Rulers are placed next to tumor after removal from body cavity to help estimate tumor size in centimeters

**Figure 5:**
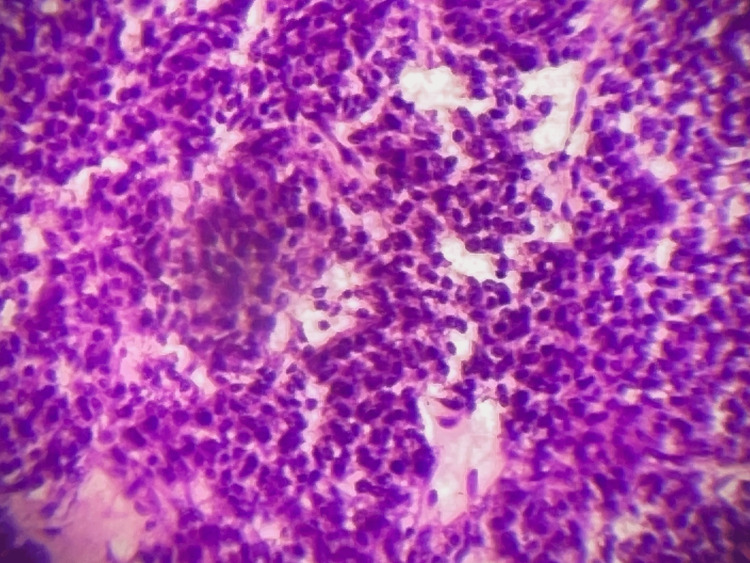
Microscopic view of specimen slide hematoxylin and eosin stain

A month after the PET scan, the patient underwent surgical excision of the mass. The enucleation of the tumor was successful, and gross images were taken both during surgery, and gross specimens following excision can be seen in Figures 3, 4. Immunohistochemistry was done on this patient’s tumor after subsequent removal. Histopathological imaging confirmed NE tumors positive for AE1/AE3, synaptophysin, cluster of differentiation 56 (CD56), chromogranin, and paired-box gene 8 (PAX8) and negative for CA19-9. Ki67 was less than 3%. The mass was located in the portal splenic groover just above the tail/neck of the pancreas. Intraoperative ultrasound showed no visible lesions and no sonographic lesions in the pancreas. The margins of resection were negative, classifying this as a grade 2 pancreatic NET. The microscopic images of specimens can be seen in Figure 5. Gastrin levels measured immediately before the surgery showed 42 pg/mL, likely this low due to octreotide and PPI use. He was discharged post-op day 6 in good condition without any complications. The patient was subsequently followed up; gastrin levels six weeks after surgery were less than 10 pg/mL. A follow-up CT and DOTATATE PET scan showed postsurgical changes and no evidence for DOTATATE-avid malignancy. Two months post tumor removal, chromogranin A levels were 108 ng/mL, with intact PTH, calcium, lactate dehydrogenase (LDH), and prolactin levels within normal limits.

A three-month gastrin level was collected, measuring serum gastrin at 10 pg/mL. PET/CT imaging revealed a mildly fluorodeoxyglucose (FDG)-avid lesion in the region of the porta hepatis, without any discrete abnormality identified in the area.

## Discussion

Depending on their hormonal output, functional NE tumors present primarily in different ways. In gastrinoma, the presentation often depends on gastrin overproduction. Studies reveal that the usual presenting symptoms of gastrinomas include abdominal pain, diarrhea, or both in 75%, 73%, and 55% of the patients, respectively, with heartburn being reported in 44% of the patients [18]. Diagnostics, while well established in theory, are sometimes muddied in clinical practice by the very treatments that are often used for symptomatic treatment.

Traditionally, the diagnosis of gastrinoma is confirmed with gastrin levels 10 times the upper limit of normal (or 1000 pg/mL) in the presence of gastric pH of less than 2 [4]. Gastrin in this patient was 231 pg/mL in 2020 and 279 pg/mL in 2021, with the normal range being between 20 and 110 pg/mL. If gastrin levels are increased but not more than 1000 pg/mL, it is recommended to follow up with a secretin stimulation test. A secretin stimulation test was not performed in this patient due to gastrin levels below the minimum threshold required, which was likely reduced by the use of PPI. PPI use was confirmed prior to the measurement of 2020 gastrin levels. Regarding the 2021 gastrin levels, there is clear documentation that the patient did indeed stop the use of PPIs two weeks prior to measurement. Postsurgical gastrin levels were obtained, and there is documented confirmation that PPI and octreotide use ceased at least two weeks prior to measurement. Gastrin levels one month status post tumor removal were 10 pg/mL, likely this low due to the remaining effects of octreotide use prior to surgery. Gastrin levels two months after surgery were 10 pg/mL.

The delay of the patient’s initial symptoms to the time of diagnosis is fairly typical of a gastrinoma masked by PPI usage. While PPIs are an established symptomatic treatment for the condition [19,20], the treatment of those same symptoms over time, without a diagnosis, has the potential to culminate in a more advanced disease state at the time of diagnosis. Studies have shown that the use of proton pump inhibitors can delay the diagnosis of gastrinoma by more than 10 years, as they can mask gastrin levels. In the present case, the symptom relief from PPI usage was not profound; however, the effect of the PPIs confounded laboratory values to the point of delaying diagnosis. The patients both with and without gastrinomas have been shown to have similar serum gastrin levels when taking PPIs [21,22],and therefore, it is reasonable to conclude that the timely diagnosis of this patient was likely delayed since PPI use was not discontinued two weeks prior to all gastrin measurements. This decision was likely made due to the increased risk of GI perforation; although normally a reasonable clinical decision, alternative measures should have been considered to allow for the accurate detection of serum gastrin levels, such as substitution with histamine 2 (H2) receptor blockers.

The best initial test for diagnosing a gastrinoma is an EGD, revealing ulcerations and thickened gastric folds with a decrease in gastric pH and an increase in basal acid output of greater than 15 mEq per hour [2,12,13]. This case report demonstrated multiple EGDs showing the inflammation and ulceration of the mucosa but never measured gastric acid levels, basal acid output levels, or documented thickened gastric folds. Basal acid output level is not commonly used in the current clinical practice due to it being invasive and time-consuming, and it unfortunately provides much discomfort for the patient. The reasoning for the lack of diagnostic testing is unclear but likely lies in the depth of bureaucracy and the inherent coordination challenges multiple providers face when simultaneously involved in the care of a singular patient.

While the diagnosis of gastrinoma is often supported with CT scan or PET scan as mentioned above, these modalities are not perfect diagnostic solutions. The sensitivity of CT scan imaging for the diagnosis of gastrinomas varies from about 29% to 51% with even lower sensitivities found for extra-pancreatic primary tumors; the overall performance of CT scans is very poor for tumors that are less than 3 cm in size. Slightly higher sensitivities are reported for the disease with concurrent liver metastases [23-26]. Partial volume averaging is another confounding factor and the potential reason that this tumor was missed on so many CT scans. Partial volume averaging occurs when tissues of different absorptions are encompassed on the same CT voxel producing beam attenuation proportional to the average value of the tissues [27].In this case report, the tumor blends into the caudate lobe and may have been obscured due to partial volume averaging. It has been estimated that up to 92% of those with gastrinomas show thickened gastric folds on EGD imaging [18,28]. In the case we reported above, the mass was missed by radiologists on numerous CT scans over the course of almost five years. Inasmuch, this case should remind clinicians to be sure to correlate the clinical picture with imaging studies to improve accuracy and reduce patient overtesting.

Somatostatin receptor scintigraphy (SRS) has a high sensitivity for assessing the extent of gastrinoma stage. SRS has become the imaging study of choice for identifying primary tumors and metastatic lesions; however, sensitivity to detect gastrinomas is dependent on the size ranging from 30% for gastrinomas smaller than 1.1 cm to 96% for gastrinomas larger than 2 cm [26,29-31]. When an octreotide scan was eventually completed on our patient, the results showed focal activity in the upper mid-abdomen correlating to a mass measuring 4.3 cm × 3.6 cm, located immediately anterior to the inferior vena cava and superior to the pancreas; this correctly identified his area of pathology; it was not until further imaging was completed that surgical treatment was completed. This, like many of the other diagnostic methods employed in our case, was delayed due to the patient’s altered presentation and laboratory results.

Serum CgA levels are a common tumor marker that is used to help diagnose and follow disease progression and treatment response. CgA levels were measured in this patient in February of 2022 with resulting levels at 359 ng/mL (N: <311 ng/mL). Subsequently, levels were measured numerous other times resulting in values ranging from 111 to 197 ng/mL. The sensitivity for CgA levels is roughly 80%, and the specificity of the test is roughly 95% [32]. Following the surgical removal of the tumor, CgA levels were normal at 108 ng/mL.

A majority of gastrinomas occur sporadically, with only ~25% of the cases due to MEN1 mutations, which are also associated with pituitary, parathyroid, and pancreatic tumors. Due to negative family history, normal calcium levels, a solitary gastrinoma, and the lack of associated clinical features, this case is believed to be sporadic in nature. A return of all tumor markers to normal levels post-excision supports this theory.

Similar to our case, the previous cases of gastrinoma diagnosis have been confounded with PPI usage creating reduced gastrin levels, which have been reported. In a case report by Wong et al. [21], a 58-year old Caucasian male presented with long-term gastroesophageal reflux disease (GERD) symptoms since the 1990s, with symptoms continuing following both H2 receptor blocker usage and PPI administration. Similar to our patient, the patient in their case had vague symptoms that recurred episodically; in contrast, their patient reported constipation, not diarrhea, and disease progression, which were much furthered in their patient compared to ours. Their case, as well as ours, serves as a reminder that PPI therapy can be both beneficial, in the way of patient symptom relief, and detrimental, in the way of delayed diagnostics and potential undetected disease progression.

## Conclusions

This case report illustrates the need for a more prompt diagnosis of gastrinomas and other NE tumors. Even if the patient does not fit the stereotypical clinical picture of an NE tumor, such as our patient’s gastrinoma, it does not indicate criteria to exclude the diagnosis from differentials when addressing recurrent symptoms. This case serves as a good reminder to all diagnostic clinicians, regarding many diseases and diagnoses. More careful evaluation of CT scans and imaging needs to be done, with the long-term clinical picture of the patient in mind. Had our patient’s neoplasm been visualized by radiologists and practitioners sooner, the outcome may have been drastically different. Additionally, had most physicians not dismissed the idea of this patient having a gastrinoma, perhaps a more prompt diagnosis could have been made, saving this patient from an immense amount of pain, suffering, and potentially unnecessary medical intervention. With this case report, we hope to not only promote a higher level of awareness of pancreatic NE tumors and the importance of quality detailed radiologic reports correlated to the clinical signs and symptoms but also remind clinicians of both the benefits and potential pitfalls of PPI use in patients with unremitting nonspecific GI symptoms of abdominal pain, nausea, vomiting, and diarrhea.

## Figures and Tables

**Figure 6:**
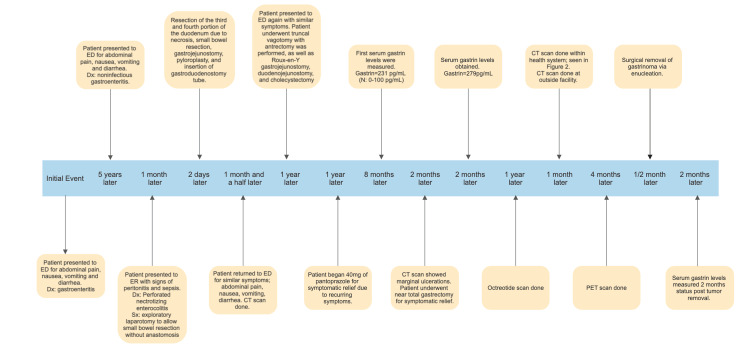
Gastrinoma with relatively low gastrin levels: a timeline of events CT, computed tomography; PET, positron emission tomography; Dx, diagnosis; Sx, symptoms; N, normal

## Appendices

Figure 6 shows the timeline of events.
